# Micro-CT and deep learning: Modern techniques and applications in insect morphology and neuroscience

**DOI:** 10.3389/finsc.2023.1016277

**Published:** 2023-01-23

**Authors:** Thorin Jonsson

**Affiliations:** Institute of Biology, Karl-Franzens-University Graz, Graz, Austria

**Keywords:** micro-CT (computed tomography), deep learning, ANN - Artificial neural networks, image segmentation - deep learning, 3D modelling

## Abstract

Advances in modern imaging and computer technologies have led to a steady rise in the use of micro-computed tomography (µCT) in many biological areas. In zoological research, this fast and non-destructive method for producing high-resolution, two- and three-dimensional images is increasingly being used for the functional analysis of the external and internal anatomy of animals. µCT is hereby no longer limited to the analysis of specific biological tissues in a medical or preclinical context but can be combined with a variety of contrast agents to study form and function of all kinds of tissues and species, from mammals and reptiles to fish and microscopic invertebrates. Concurrently, advances in the field of artificial intelligence, especially in deep learning, have revolutionised computer vision and facilitated the automatic, fast and ever more accurate analysis of two- and three-dimensional image datasets. Here, I want to give a brief overview of both micro-computed tomography and deep learning and present their recent applications, especially within the field of insect science. Furthermore, the combination of both approaches to investigate neural tissues and the resulting potential for the analysis of insect sensory systems, from receptor structures *via* neuronal pathways to the brain, are discussed.

## Introduction

Technological advances during the last decades have given researchers many new tools and methods that either opened up hitherto less approachable or even inaccessible areas of science or that enabled pushing the boundaries of already established fields. Some of the most obvious advances have taken place in the development of computer technology, with computing power and available space to store and access information increasing near exponentially over the last 50 years (1). This computational revolution has driven the development and rise of, amongst many others, advanced imaging technologies as well as artificial intelligence (AI) with its subfields machine and deep learning. Here, I present an overview of a (necessarily small) selection of the recent advances in those quickly expanding fields, with a focus on X-ray micro-computed tomography (µCT) and AI-supported approaches to analyse and interpret two- and three-dimensional (2D and 3D, respectively) image data gathered with µCT. For both fields, I will give a brief introduction and illustrate how these technologies and methods can be combined specifically to shed light on the external and internal morphology of insects; their outer form as well as their neuronal structure.

## X-ray micro-computed tomography

### Introduction

Since the development of the first X-ray computed tomography (CT) scanner by Godfrey Hounsfield in the early 1970s (2), CT scanners have become practically ubiquitous in hospitals around the world for the fast, non-invasive detection of a wide range of pathologies, from brain aneurysms to bone fractures and tumours. With the advent of ever cheaper scan units and increasing image resolutions, CT has also become an increasingly interesting tool for biologists to investigate the external and internal morphology of their organisms of interest.

Many excellent reviews exist that describe the development, function and the many uses of CT and µCT in both the medical and natural sciences in detail (e.g., 2–9). Thus, the method will be explained only briefly here: Generally, in commercially available µCT scanners used in laboratories (also called desktop scanners), X-rays are generated through the deceleration of fast electrons in an X-ray tube and directed in a cone-shaped beam towards a sample placed on a rotating stage in front of a 2D detector array. Traversing the sample, X-rays are attenuated, with the amount of attenuation or absorption depending on electron density and atomic number of the chemical elements in the sample (10). The detector is collecting the attenuated X-rays in form of a 2D X-ray image, called radiograph. The sample is then rotated in small angular intervals and an image is taken at each step, resulting in datasets of many 100s or 1000s of radiographs. Using filtered back projection or iterative reconstruction algorithms (7, 11), 2D tomographic images (axial cross-sections or ortho-slices) of the whole sample are generated, which form the basis for a complete 3D reconstruction, where the grey value of each voxel represents the X-ray attenuation of this volume element of the sample. Both 2D tomographic images and 3D reconstructions can then be used to explore the interior and exterior structures of the sample qualitatively and quantitatively.

The main advantage of µCT over classical techniques for anatomical and morphological studies is certainly the possibility to non-destructively and non-invasively create whole-volume 3D reconstructions of complete organisms in a relatively short amount of time due to minimal sample preparation, fast image acquisition and streamlined data post-processing (12). This is in sharp contrast to sectional image reconstruction using time-consuming and destructive histological methods including complex staining and embedding protocols, microtome slicing, manual mounting and sequential imaging of samples with light microscopes (LM) or confocal laser scanning microscopy (CLSM). Disadvantages, on the other side, are the comparatively lower image resolution of most µCT scanners in comparison with CLSM and LM (typically ~200 nm; the Abbe diffraction limit. 13) and a lack of contrast in soft tissues. The latter is due to the low absorption rates of X-rays in biological tissues containing predominantly low-Z elements (low atomic number; e.g., carbon, oxygen, nitrogen), leading to mostly uniform density patterns in the image data (10, 14, 15). To overcome this issue, various contrasting or staining agents can be used to increase tissue contrast. Contrasting agents are usually comprised of high-Z elements that bind to tissues, increasing the X-ray absorption and thus image contrast. Common staining methods include elements like iodine (e.g., Lugol’s iodine potassium iodide solution), tungsten (phosphotungstic acid; PTA), osmium (osmium tetroxide), lead (lead nitrate or lead acetate) or silver (Bodian’s reduced silver stain or Golgi stain). The reader is here referred to 10, 15–17 for in-depth evaluations of contrast-enhancing solutions and methods. Most of these contrast agents are relatively non-specific stains. For example, iodine is generally absorbed by lipid-rich soft tissue, PTA binds to various proteins like fibrin and collagen and Golgi’s method stains neurons randomly (10, 15). To improve selectivity of contrast-enhancing stains suitable for µCT, recent studies have employed immunohistochemical methods, coupling neuron-specific antibodies with gold nanoparticles to selectively stain neuronal nuclei in mice (9, 18). This approach of combining traditional immunohistological staining protocols with X-ray-absorbing nanoparticles is still new but has the potential to substantially improve the quantitative analysis of nervous systems using µCT.

Another way to produce high-contrast images and 3D models of soft and nervous tissue is to use phase-contrast µCT (PC-µCT). X-ray beams are not only absorbed when passing through a material but the electromagnetic waves also undergo phase-shifts at the boundaries of materials with different densities and thus refractive indices. Phase shifts along material boundaries can be analysed and used to reconstruct images with high edge-contrast between tissues without the need for contrast agents. To detect these changes in phase, parallel and highly coherent X-ray beams are needed, so that the use of phase-contrast µCT is usually constricted to synchrotron beamlines (15, 19, 20; however, see 21–23 for notable exceptions). Although this makes synchrotron-based PC-µCT as a technique not as accessible as general absorption-contrast µCT using laboratory-based desktop scanners, there are certain advantages to consider: Maximum spatial resolution using PC-µCT is considerably increased, reaching 10-50 nm/voxel, depending on the sample dimensions (and often at the cost of a highly reduced field of view; 15, 22, 24). Additionally, image acquisition at synchrotron facilities can be faster by a factor of 1000 when compared to commercially available µCT scanners, allowing for both high sample throughput and fast collection of time-series scans (9, 15, 25; and see examples below). In Walker et al., e.g., the authors present time-resolved (sub-millisecond) tomographic data showing the mechanics and kinematics of the blowfly (*Calliphora vicina*) thoracic flight motor in 3D during tethered flight at a resolution of ~3 µm/voxel (26).

### µCT and insect neuroscience

While the early generation of medical CT scanners only allowed identifying rather large and well delineated anatomical structures like brain tumours, major blood vessels (for angiography) or bones (for fracture detection and density measurements), a steady improvement of scanning speeds and spatial resolution led to an increased use of CT in small-mammal preclinical studies (mostly rodents; *in* and *ex vivo*; 3, 4, 6). The comparably low resolution of conventional medical CTs prevented the widespread use of this technique for invertebrate morphology. From the beginning of the 2000s, however, µCT with spatial resolutions down to ~5 µm became more widely available (and affordable), leading to a “renaissance of insect morphology” in the years to come (27, 28). Today, many desktop µCT scanners can routinely achieve isotropical resolutions down to 1-2 µm/voxel, with some commercially available high-end scanners now even approaching nanoscale resolutions of ~0.4-0.2 µm/voxel (8, 29, 30). Additionally, recent years have also seen advances in the development of laboratory-based phase-contrast CT scanners with resolutions in the range of 0.5-0.1 µm, approaching spatial resolutions available at synchrotron beamlines (21, 22).

These advances in µCT technology have made it feasible to not only use CT as a tool in insect morphology (e.g., larval development: 31, 32; locomotion: 25, 33; muscular system: 19, 34; respiration: 35, 36; vision: 37, 38; wing structure: 39, 40) but to also explore the central nervous system (CNS) of insects. In 2007, for example, Mizutani et al. imaged the brain of larval *Drosophila melanogaster* using synchrotron-based µCT with a resolution of ~1 µm/voxel. The authors could visualise the major components of the developing supraoesophageal ganglion, including the optic lobes and the peduncles of the mushroom bodies. 3D views of the brain also included neuronal cell bodies and axons (41). A year later, Ribi et al. (42), presented the first application of a lab-based µCT-system for the scanning and 3D visualisation of an invertebrate brain. Here, the authors scanned whole heads of the honey bee *Apis mellifera* stained with osmium tetroxide at a resolution of 7 µm/voxel. In the resulting 3D reconstruction, the main brain neuropiles (protocerebrum, antennal and optic lobes as well as the mushroom bodies and some substructures) are visualised and well delineated, demonstrating the suitability of the method for insect neuroscience (42). In 16, 3D and 2D views of a lacewing head stained with iodine and a mantophasmid tibia stained with PTA and scanned with a lab-based µCT are presented at even lower resolutions of 2 and 0.9 µm, respectively. In the lacewing head, individual ommatidia and the layers of the optic lobe (lamina, medulla, lobula) are clearly delineated, as are other brain neuropiles like the peduncles and calyces of the mushroom bodies. In the 3D model of the tibia, some individual sensory cells as well as a scolopidial organ can be distinguished (16). Building on the pioneering work by Ribi et al. (42) Smith et al. (43) refined and adapted PTA-staining procedures to stain the brains of 19 bumblebees (*Bombus terrestris*) that were subsequently µCT-scanned at resolutions ranging from 3.1-4.6 µm/voxel. The resulting 3D reconstructions were then used for the first time to quantify brain allometry and differences in the volumes of bee brain neuropiles (43). Expanding on this work, Smith et al. (44) used their existing protocols to scan and segment the brains of 78 adult bumblebee workers exposed to either a neonicotinoid insecticide during development or to a control sucrose solution. Performing volumetric analyses on the brain neuropiles, the authors could show that the mushroom body calyxes of workers exposed to the insecticide had significantly lower relative volumes compared to the control bees and that this negatively influenced their behaviour in learning and responsiveness tests. Rother et al. (45) then reused parts of the data collected by Smith et al. (44) to construct the first 3D insect brain atlas based on µCT instead of on CLSM data. The atlas visualises all major brain neuropiles in *B. terrestris*, including smaller ones like the central complex and its sub-divisions, the anterior optic tubercles and substructures of the mushroom body calyces. Additionally, several prominently visible neuronal tracts linking different neuropiles are identified. Although the authors also map some individual neurons and their projections into the central complex, these are visualised through classical immunostaining methods and CLSM, as no individual neurons are visible on the CT data itself (45). In contrast to this, Ribi and Zeil (46) successfully traced the origins and projection areas of all large ocellar interneurons (L-neurons) in orchid bee (*Euglossa imperialis*) brains stained with osmium tetroxide and µCT scanned at a resolution of 3 µm/voxel. Using fibre-tracing methods, the authors reconstruct the individual dendritic origins of the 27 L-neurons in the ocellar plexi (below the retinae) and follow their paths to the ipsi- or contralateral protocerebrum where they terminate within close proximity to optic (lobula) and mechanosensory (antennal lobe) interneurons. While identification and reconstruction of the large fibers (up to 22 µm in diameter) and their main arborizations proves unproblematic at the resolution used, the authors also acknowledge that fine arborizations and branching patterns, and thus exact connectivities, are not resolved (46).

Through these examples, it becomes clear that a combination of adequate staining protocols and voxel resolutions in the low µm-range is sufficient to gain a general insight into the organisation of the insect CNS (end even some of its functions; 44), at least on the level of brain neuropiles. At resolutions of ~1-3 µm/voxel (now achievable with many desktop µCTs), cell bodies and axons of some neurons are visible, but these most likely constitute only a subset of the neuronal population, i.e., neurons with sufficiently large cell bodies and axon diameters to enable a clear distinction from background tissue. While identification and tracing of populations of larger neurons is possible, reconstructing their finer dendritic arborization or synaptic fields would certainly require higher resolutions (as demonstrated below).

Overall, neuron sizes are highly variable in both vertebrates and invertebrates but are generally smaller in smaller animals (47, 48). In insects, neuron cell bodies can reach diameters of many 10s of µm while the minimum soma size seems to be limited to ~2-3 µm due to limitations in the size of the nucleus (49, 50). The diameter of unmyelinated axons, on the other side, only seem to be constrained by the electrical properties of the neuronal cell machinery, the ion channels. At diameters <0.1 µm, the stochastical noise generated by thermodynamically activated ion channels increases exponentially, preventing meaningful information transport *via* the axon (51, 52). Taking neuron cell body and axon sizes into account, Chin et al. (24) argue that a voxel resolution of 0.3 µm “…is not just a sufficient level but in fact an optimized compromise between…” image resolution and acquisition speed when setting out to map neuronal brain architecture in insects. In the same study, the authors then use synchrotron-based µCT at said resolution and 250 Golgi-stained *Drosophila melanogaster* heads to create a standard 3D map of the fly’s whole-brain neural network “…in a few days” (24).

This study exemplifies the potential of modern µCT technology for the field of insect morphology and neuroscience: Non-destructive, fast, high-resolution data acquisition at levels sufficient to enable the construction of three-dimensional, detailed outer and inner anatomy models in conjunction with maps of the neuronal architecture. As a result, µCT could be easily applied to study insect sensory systems in high anatomical and neuronal detail: high-resolution scans of stained whole-body preparations can be generated and the sensory system in question can be visualised, virtually dissected and explored; from the outer signal receiving structures to the sensory receptors to the neuronal projections into the brain or other body parts. Arguably, resolution of fine axonal projections or dendritic arborizations will not be as high as with CLSM, but both synchrotron- as well as modern lab-based CT setups can achieve resolutions high enough (~0.3 µm) that such quick models can provide valuable overviews and inform and guide subsequent experimental approaches using other, complementary methods to add to our understanding of the sensory systems in question.

## AI, artificial neural networks and deep learning

After performing high-resolution µCT scans, one is confronted with very large datasets, usually consisting of many hundreds or thousands of images with megapixel resolution. A µCT dataset of a bush-cricket ear, for example, can thus easily contain 3 gigabytes (GB) of raw (8 bit; uncompressed) image data at 1 µm/voxel resolution (*C. gorgonensis*, ~1.5 x 1.5 x 1.3 mm³; 53). The much smaller head of a *Drosophila* (~0.5³ mm³) scanned at a voxel resolution of 0.3 µm – as carried out by 24 – would result in a ~4.6 GB dataset per individual fly. To reconstruct the outer and inner anatomy of an organism, the image data has to be labelled or annotated according to the desired level of detail, a process called segmentation. In essence, during segmentation each voxel of interest in a 3D dataset is assigned a label that identifies the voxel belonging to a specific structure or object and there are now many free, open-source (e.g., 3D Slicer, Drishti, ITK-SNAP, SPIERS) as well as commercial (Amira/Avizo, Dragonfly, VGStudio) software packages that allow importing, manipulating and segmentation of 3D datasets (see e.g., 7, 54, 55). Some segmentation tasks can be performed in a semi-automated manner, especially when contrast between the structures to be labelled and image signal-to-noise ratio is high. The outer cuticle or mandibular structures of insects, for example, can often be easily segmented by simple thresholding methods, as these relatively hard body parts will be clearly delineated from softer materials. For the most part, however, segmentation of µCT data is a time-intensive process that often involves hours or days of manually labelling images.

While it is feasible to manually segment e.g. the cuticle and major internal organs of one or several CT-scanned specimens (56, 57), the cost in terms of time quickly becomes prohibitive when one wants to investigate species on a population level (24, 44, 45) or when attempting to segment neuronal networks (58, 59). For the latter cases, AI techniques like deep learning using artificial neural networks (ANN) can provide solutions to enable the analysis of large-scale CT datasets within sensible timeframes.

There are a multitude of introductory textbooks (e.g., 60–63) and excellent reviews about deep learning, its history (64 recapitulates 70 years of ANN development) and implementation in both biological and medical contexts (e.g., 65–70). Here, I will only give a brief overview before concentrating on examples of specific network architectures applied to (mostly) biological and biomedical imaging, and potential applications of deep learning specific to µCT data in insect science.

### A brief introduction to deep learning

Deep learning, a subset of AI machine-learning techniques, has experienced considerable breakthroughs in the last decade that makes it a valuable tool for practically anyone who wishes to analyse very large and complex datasets (“big data”); from engineers constructing self-driving cars to economists predicting stock-market developments to linguists performing natural language processing and speech recognition tasks to molecular biologists investigating protein folding structures (71, 72).

The advantage of deep learning over more general machine-learning approaches lies in the organisation of the specific ANNs employed to solve particular tasks. In conventional machine learning, an algorithm learns to perform a certain task on the basis of manually designed (“hand-crafted”) features that have to be extracted from the raw data (67, 68). This approach thus requires a good *a priori* understanding of which feature representations of a given dataset can be useful for solving the task at hand. In contrast, ANNs don’t rely on a manual definition of features but can learn abstract hierarchical features directly from the raw data (65, 69). In supervised learning, the ANN is given pre-labelled training data, presenting the ground-truth for a specific task. Given enough training data, the network then learns to predict the correct label for data is has not encountered in the training phase. In unsupervised learning, the network uses unlabelled data to uncover (often abstract) features or patterns inherent in the data. Such networks are often used in combination with supervised ANNs to enhance the overall performance (67, 73, 74).

ANNs are composed of various layers of artificial neurons or nodes (loosely inspired by the organisation and connectedness of neurons in the brain) that take information from previous layer neurons, apply mathematical functions and associated weights and biases to it and pass it on to neurons in the next layer of the network. A basic ANN normally consists of an input layer, a (low) number of hidden neuron layers that are sequentially (often fully) connected and an output that contains the result (e.g., a decision or classification). Convolutional neural networks (CNN) are a class of ANN that are especially useful for the analysis of 2D data like images or sequential data and are extensively used for tasks like image recognition, object detection, motion tracking or speech analysis. Here, the first layers of the network after the input – the convolutional layers – are inspired by the neuronal organisation of the visual cortex, so that neurons are not fully connected to each other but form receptive fields with shared weights and biases (60, 73, 75). Subsequently, the information extracted from the convolutional layers is sent to a number of pooling layers, which are simplifying the information before sending it to either more convolutional layers or fully-connected layers and finally to an output (65, 68). While the overall architecture of CNNs seems to be more complex than that of standard ANNs with only a few hidden layers, the sharing of weights and biases in the convolutional layers and the subsequent pooling reduces the overall number of connections and parameters, making CNNs easier to train (76).

While CNNs with various but usually low depths were already in use in the 1990s and 2000s (64, 77), it was the development and implementation of ever faster graphics card processors in conjunction with advances in CNN architecture that led to important breakthroughs for deep learning in computer vision in the early 2010s. In 2013, Krizhevsky et al. presented a 13-layer deep CNN (AlexNet; five convolutional layers interspersed with five pooling and normalisation layers plus three fully connected layers) which classified the 1.2 million images of the ImageNet dataset into its 1000 individual classes with nearly half the error rates of the then state-of-the-art models (70, 76). Later research on transfer-learning (the idea that networks pre-trained on bigger, more general datasets could be used as basis for fine-tuned networks performing different but related tasks; 78, 79) and deep residual learning networks resulted in the construction of very deep ResNets (up to 152 layers) that further significantly reduced error rates in image classification, detection, localisation and segmentation tasks (80). ResNets are now often used as backbone architecture for the development of other networks for computer vision tasks (81). Prominent examples are the fully convolutional networks U-Net and V-Net, both building on the ideas of ResNets to increase performance specifically in 2D and 3D biomedical image segmentation (67, 82, 83). The next section will mainly concentrate on these more recent deep learning networks and on their use in the context of image segmentation. Since the (bio)medical sciences have not only been playing a considerable role in the development of CT and µCT technology but have also partly driven the development of ANNs able to analyse the resulting data, examples given will include implementations from biological and medical fields alike.

### AI-supported image analysis

In the medical sciences, mainly driven by the early and ubiquitous application of imaging devices like MRI and CT as diagnostic tools, AI has been used since the 1990s on an ever-growing scale (see e.g., 77, 84). Here, learning algorithms are used in image analysis tasks ranging from object detection and classification (e.g., nuclei, cell types, tumours) to automated segmentation in 2D and 3D (e.g., brain, liver, prostate, cardiac vessels; 65, 70, 82, 83). The applications for AI in medical imaging are numerous, as are the data sources used to train the networks on, which leads to a high amount of very specialised ANN implementations (and associated issues concerning training data availability, label noise, non-standard data acquisition with different modalities, biased datasets, etc.). Some examples of these implementations for the analysis of medical CT data are given below:

Hamwood et al. (85) use two modified U-Nets to automatically segment the boundary of the bony parts of the human orbit from MRI or CT scans with results similar to segmentation by human specialists, although in a fraction of the time (1.5-3 min instead of ~4 h). However, the training and test data only includes MRI and CT scans from 11 test subjects, suggesting that the algorithm could perform better after being trained with more data and also highlighting the problem of data availability in some clinical contexts (85). In other circumstances, however, data availability is less of an issue: Xie et al. (86), for example, use publicly available, high-resolution (sub-mm in this context) lung CT scans from 5000 subjects with chronic obstructive pulmonary disease (COPD) to train a relational 2-stage U-Net (RTSU-Net) to automatically segment the five pulmonary lobes. Their approach includes one stage that extracts global features from the whole 3D scan and a second (simultaneous) stage that captures local, high-resolution details. The authors then apply a transfer learning approach and retrain the network with CT scans of the lungs of 370 patients with suspicion of COVID-19. In both cases (COPD and COVID-19), RTSU-Net significantly outperforms three standard networks and reaches human segmentation accuracy, even for the COVID-19 dataset with its much smaller training set and different pathologies (86).

For a different segmentation task, Lindgren Belal et al. (87) construct and train a fully convolutional network to automatically identify and measure the volumes of 49 bones of the human skeleton (mainly vertebrae and ribs) on the basis of whole-body CT scans. The CNN is trained on 100 manually segmented CT scans, tested on scans of 46 different patients and its performance compared against segmentation results of a trained radiologist. This specialist manually segmented a set of bones twice for five patients with data from two different CT scans per patient. Dice coefficients (a measure of accuracy that considers true positive, false positive and false negative labels; with a value of 1 corresponding to full and 0 to no overlap of automated segmentation and ground-truth) are in the range of 0.85 but, interestingly, the intraindividual volume differences between bones of the five test-patient scans are much higher for the manual observer (3-14%) than for the CNN (1-7%), suggesting that the CNN produced results with a much higher reproducibility than the human specialist (87).

More in the context of preclinical µCT using small mammals, Malimban et al. (88) apply the nnU-Net pipeline (an “out-of-the-box”, hyper-versatile, self-configuring segmentation method based on the U-Net architecture; 89) to an auto-contouring and -segmentation task of internal organs of mice on the basis of *in vivo* µCT images. The nnU-Net is applied to the 3D data and shown to perform better and more robustly than a comparable 2D network (Dice coefficients of 0.91-0.97, depending on segmented organ). Additionally, the authors show that the nnU-Net’s performance on data taken from a different distribution than the original training data (contrast-enhanced instead of normal CT scans) is far superior to the 2D network, demonstrating a high generalisability of the trained network (88).

In biology, the use of AI to analyse image data has been on the rise as well, especially since the advent of deep CNNs and the accompanying frameworks (e.g., Keras, TensorFlow) which dramatically decreased the error rates of ANNs and increased the facility of implementing specific model architectures (i.e., the structure of the neural network).Widespread uses for AI in biological image analysis are the automated identification of species from still images (e.g., iNaturalist, Merlin Bird ID or Flora Incognita; 90) or markerless pose estimation or motion tracking from videos of behaving animals (e.g., DeepLabCut, 91, 92), to name but a few examples. There are certainly many more uses of deep learning in ecology, bioacoustics or behavioural sciences and the interested reader is referred to e.g., 66, 93–97.

### ANNs and insect neuroscience

While deep learning networks in species recognition or motion tracking perform mainly object recognition tasks, ANNs in neurobiology are now increasingly used to perform segmentation of neurons and biological neural networks, usually based on 3D microscopy data. Here, data stems from using CLSM, scanning electron microscopy (EM), transmission EM or similar high-resolution imaging methods. Li et al., for example, apply a complex, 200+ layer, ResNet-like CNN to automatically trace neurons from 3D optical microscopy datasets of single (stained) neurons of various vertebrate and invertebrate species. The authors demonstrate that their neural segmentation approach is robust against image noise and that it significantly outperforms prior state-of-the-art tracing methods (98).

In another approach, Januszewski et al. (59) construct a new type of flood-filling network (FFN) to automatically trace and reconstruct 3D neurons in serial block-face EM images of a section of zebra finch brain (~100³ µm³ at a xy-resolution of 9 nm and z-resolution of 20 nm; containing ~450 somata). While the network itself is a 19-layer CNN building on AlexNet and ResNet characteristics, it differs from these in that it only segments one object at any given time and that it implements a new type of feedback pathway. This feedback enables the network to use information about previously labelled voxels, increasing its performance by an order of magnitude when compared to two baseline CNNs (1.1 mm mean error-free neurite length and only four wrongly merged neuronal processes within 97 mm of traced neurons). Applied to different EM datasets of *Drosophila* optic lobe and mouse somatosensory cortex, the FFN outperformed all previously applied methods, including a U-Net approach (59). With the help of this FFN and using focussed ion beam SEM, Scheffer et al. (99) built the complete connectome of a *Drosophila* hemibrain (one half of the central region of the brain, including the whole central complex; ~250³ µm³ in volume with a resolution of 8 nm/voxel), identifying, tracing and characterising ~25000 individual neurons, their neurites and synapses (~20 million). This connectome represents approximately 1/4 of all neurons in the fly’s brain and substantially adds to information previously collected in smaller sections of the *Drosophila* brain using similar methods (e.g., the connectome of the ~1000 neurones of the mushroom body α lobe; 100). However, even with the extensive use of ANNs to analyse and segment the data, the authors estimate that an additional 50-100 person-years of proofreading were invested to generate the final outcome (99).

Despite these advances in using AI and deep learning tools for automated analysis and segmentation tasks, there seems to be only two studies so far that investigate fully-automated tissue segmentation from µCT data in a non-medical context: Toulkeridou et al. (101) produce complete µCT scans of 76 species of ants (iodine-stained; resolutions between 0.5 and 3.5 µm/voxel) and subsequently segment their brains in a semi-automated manner (using a seed-based watershed method followed by manual post-processing; ~5 hours work per brain). This provides the authors with a decent training (60%) and testing (40%) dataset of 76 brains with an average of 1000 labelled images (cross-sections in all three dimensions) each. They then train a 15-layer U-Net on the 2D data (using data from all three dimensions) to segment the brain from other tissue (fat, muscles, etc.) and the cuticle. The trained ANN segments whole brains in 1-2 minutes, achieves Dice coefficients of 0.9 (after combining data into 3D and post-processing) and is shown to also segment the brains of other insects (wasp and praying mantis) with similar accuracy (101). However, a significant improvement of its performance could potentially be accomplished if the network was adapted and trained on 3D data, thus taking spatial or structural information like position and size into account (like ANNs in 59, 88).

To this effect, in a recent study by Lösel et al. (102), a 3D U-Net is trained to automatically segment µCT data of brains of both honey bees and bumblebees (PTA-stained; 5.4 µm/voxel resolution) into six major areas (antennal lobes, mushroom bodies, central complex, medulla, lobula and ‘other neuropils’). Here, the authors make use of the free, open-source, online platform Biomedisa (biomedisa.org; 103) for semi-automated and deep learning-supported image segmentation. In its basic form, Biomedisa allows the user to upload a sparsely annotated 3D dataset (i.e., structures of interest are not completely labelled but labels are supplied in intervals of tens of slices) in a variety of common file formats. The pre-segmented images are then used as starting points for adaptive weighted random walks algorithms that perform ‘smart interpolation’ whilst taking into account the underlying 3D data (103). Using this semi-automated way, one can generate complete sets of segmentation labels of 3D datasets in a fraction of the time usually needed and with better accuracy when compared to other interpolation methods. Lösel et al. (102), thus create a training dataset of 26 fully labelled honey bee brains with an average Dice score of 0.97 when compared to manual labelling and in contrast to a Dice score of 0.93 when using standard interpolation methods (see also 103, for a table comparing various segmentation methods). The authors proceed to use Biomedisa’s inbuilt 3D U-Net architecture to train a network that subsequently segmented a further 84 honey bee and 64 bumblebee brains with Dice scores of 0.99 and 0.98, respectively. This particular approach allowed the authors to perform quantitative neuroanatomical comparisons of brain areas for not a select few, but a high amount of individual animals in two species whilst reducing manual segmentation and analysis effort by up to 98% (102).

## Conclusion

Overall, the studies by Toulkeridou et al. (101) and Lösel et al. (102) and, likewise, the lack of other research in this direction, highlights the following: While using deep learning approaches for automated segmentation tasks in insects (or invertebrates in general) is certainly possible, one of the bottlenecks is still the availability of large sets of standardised and sufficiently labelled training data. Although, projects like Biomedisa and the resulting (openly available) data present ways to overcome this issue (102, 103). However, while more and more researchers are implementing µCT in their studies, datasets like those presented above (24, 44, 45, 101, 102), where either many closely related species or many individuals of one species are imaged with the same modality, are few and far between. While medical researchers can often access more or less standardised image data from big, centralised databases (86), similar options are usually not (yet) available for biologists. Nevertheless, the increasing ubiquity of µCT in biology and the efforts of the community to share raw and annotated data on open-access repositories and websites (e.g. Dryad, Zenodo, Biomedisa) and pre-trained networks on developer platforms (e.g., GitHub) provide interesting possibilities: Researchers could make use of transfer learning methods and use those pre-trained networks in conjunction with own training data, augmented datasets and parameter fine-tuning to derive ANNs able to better fulfil a desired task (78, 79). Conceivably, one could also collect more diverse datasets and train deep ANNs with advanced architectures (like the promising nnU-Net used in 88 or FFNs in 99) to extract features that will allow them to perform tasks independent of variables like imaging modality, resolution, staining protocol or species. Hypothetically, such a network could then be trained to, for example, automatically segment and quantify not only whole insect brains, but also individual neuropiles, across taxa or during various developmental stages.

Also, while image resolutions like those attained with EM methods (59, 99) are far higher than those of state-of-the-art lab-based µCT scanners, they are, in practice, too high to even allow imaging of whole invertebrate brains in a realistic timeframe. For example, Scheffer et al. (99) report the total imaging time for one (!) *Drosophila* hemibrain as “roughly four [ … ] years”. However, using either synchrotron or lab-based CT methods in combination with appropriate tissue fixation and staining protocols to create sufficient tissue contrast, imaging the neural tissue of complete insect brains or even whole bodies at sub-µm resolution (0.3 µm/voxel, as suggested by 24) has become a, if not trivial, then at least not unreasonable endeavour. Such datasets would undoubtedly be quite large: a whole-body *Drosophila* (which very roughly fits a bounding box of 3.5 x 1.5 x 1.5 mm³), scanned at 0.5 µm/voxel resolution, would result in ~63 GB of uncompressed image data. However, employing an ANN that analyses data in 3D and has been trained to, e.g., separate neuronal tissue from internal organs and muscles, a dataset like this could be automatically segmented into broad anatomical components in a matter of minutes. If one could also conjugate contrast-enhancing nanoparticles to antibodies specifically targeting insect neurons (e.g., anti-horseradish peroxidase antibodies; 104, 105), like it has been shown in mice (18), an additional ANN could subsequently identify and trace those targeted neurons. While it can be argued that even a resolution of 0.3 µm/voxel combined with targeted contrast enhancers is still not sufficient to trace the finest dendritic arborizations and thus the full connectivity of neurons (as done in 99, 100) such an approach would still significantly facilitate the exploration of neuronal circuits in the insect CNS.

In summary, combining state-of-the-art µCT scanning methods with subsequent, AI-supported, 3D data analysis to study insect morphology has the potential to allow researchers to approach certain questions or problems from a different methodological angle: µCT can be used to quickly, cheaply, non-invasively and non-destructively image either whole insects or specific regions of interest with relatively high spatial resolution. Resulting datasets can be used to produce highly detailed virtual 3D models of the animal’s exterior and interior that can be virtually explored and dissected. These models already allow for the qualitative and quantitative analyses of major anatomical structures that would be technically highly complicated and very time-consuming to carry out with more traditional methods: One can, for example, easily compare the relative densities of various parts of the chitinous cuticle (intra-, inter-individually or between species), set morphological landmarks and perform morphometric measurements in 3D with high accuracy or measure the volume of internal organs, trachea, muscles or parts of the CNS. As an aside, these models can additionally serve as detailed geometrical blue-prints for the construction of virtual sensory receptors like, e.g., mechano- or electroceptive hairs (106, 107), the different antennal structures linked to the mechanoreceptive Johnston’s organ (108) or the tympanic auditory receptors of bush-crickets (109). On the basis of these 3D geometries, advanced finite element models (another quickly advancing field with great potential that could only be mentioned briefly here) can then be constructed to simulate the structural mechanical behaviour in response to physical stimuli, be it mechanical motion, acoustic waves or electric fields; thus also complementing our understanding of insect sensory systems. Finally, there is the possibility of implementing properly trained, deep ANNs to automatically classify the vast number of digital pixels resulting from high-resolution µCT scans into categories like cuticle, muscle or neuronal tissue, leading to the automated, fast and accurate segmentation and tracing of the internal insect anatomy, including at the level of individual neurons. Although some problems do still exist – like standardised training data availability or neuron-specific contrast-enhancers – these will most likely be solved in the near future, leading hopefully to methodological frameworks and streamlined pipelines that will allow researchers to, for example, map whole sensory systems, from the receptor, *via* its neuronal pathways, to the brain. Such projects could be completed in weeks or a few months, instead of taking years, thus helping in extending our knowledge and understanding of the neuronal systems guiding the behaviour of insects.

## Author contributions

TJ devised and wrote the manuscript. The author confirms being the sole contributor of this work and has approved it for publication.
